# The extent of inflammatory infiltration in primary cancer tissues is associated with lymphomagenesis in immunodeficient mice

**DOI:** 10.1038/srep09447

**Published:** 2015-03-30

**Authors:** Lianhai Zhang, Yiqiang Liu, Xiaohong Wang, Zhiyu Tang, Shuangxi Li, Ying Hu, Xianglong Zong, Xiaojiang Wu, Zhaode Bu, Aiwen Wu, Ziyu Li, Zhongwu Li, Xiaozheng Huang, Ling Jia, Qiang Kang, Yong Liu, David Sutton, Lai Wang, Lusong Luo, Jiafu Ji

**Affiliations:** 1 Key laboratory of Carcinogenesis and Translational Research (Ministry of Education), Department of Surgery, Peking University Cancer Hospital and Institute, Beijing, China; 2 Department of Pathology, Peking University Cancer Hospital and Institute, Beijing, China; 3 BeiGene (Beijing) Co. Ltd, No. 30 Science Park Road, Life Science Park, Changping District, Beijing, China

## Abstract

Xenotransplantation of human cancers into immunodeficient mice is a very useful approach for studying human tumor biology. However, the occasional occurrence of lymphomagenesis in some mice can spoil the model and must be investigated in detail. We found that a high percentage (32.5%, 26/80) of cancer patient-derived xenografts (PDXs) resembled lymphoma in NOD/SCID mice. Of the 26 xenografts, 23 were human-derived expressing human CD45 (hCD45+) and proved to be of the B-cell subtype (CD3-/CD20+), and they were all positive for Epstein - Barr virus (EBV). The remaining 3 xenografts proved to be mouse-derived for both hCD45- and negative amplification of a human gene. The most interesting finding is that gastric cancer had much higher rates (24/126, 19.0%) of lymphoma formation in the PDX model than did colorectal cancer (1/43, 2.3%). Statistical analysis revealed that cancer type and inflammation in the parent tumor are significantly associated with lymphomagenesis. Further validation discovered lymphomagenesis by inoculating only gastritis mucosa. Therefore, our findings suggest that it is necessary to take precautions when directly xenografting cancer tissues with remarkable baseline inflammation, such as gastric cancer into immunodeficient NOD/SCID strains. Further, the established xenograft models should be validated by both leukocyte markers and human gene signatures.

Patient-derived tumor xenografts (PDXs), which are established through the xenotransplantation of human cancers into immunodeficient mice, are able to mirror patients' histopathological and genetic profiles^1^,^2^,^3^,^4^ and are very useful for studying human tumor biology. There has been a surge in the use of these experimental models to predict the clinical activity of anti-cancer therapies and discover predictive biomarkers^5^,^6^,^7^. The most commonly utilized mouse strains, such as the nonobese diabetic severe combined immunodeficiency (NOD/SCID) strains, are deficient in both innate and adaptive immunity and thereby permit the high engraftment rate of human tumor tissues^8^,^9^,^10^.

The SCID mouse model, however, has several pitfalls. Xenografting primary human solid tumor tissue into immunodeficient mice may fail to induce lymphoma using highly immunodeficient NOD/SCID mice. In the early 1990s, researchers had discovered that B cell lymphomas occurred later in a large percentage of SCID mice that received peripheral blood lymphocytes (PBL) from individuals infected with Epstein-Barr virus (EBV), particularly after the transfer of lymphocytes by intraperitoneal injection^11^,^12^. Additionally, in two recent studies, researchers found that after xenografting primary human hepatocellular carcinoma (HCC) and non-small cell lung carcinoma (NSCLC) tumor fragments into NOD/SCID or NSG immunodeficient mice (NOD/SCID/interleukin 2 receptor gamma chain null strains), a high percentage (11 of 21 in HCC, and 19 of 153 in NSCLC) were found to be human B lymphomas^13^,^14^.

In this study, through the systematic establishment of a larger panel of xenograft models for pre-clinical drug testing, we discovered a high probability of lymphoma formation when implanting human tumor tissues into immunodeficient NOD/SCID mice, particularly for gastric cancer. The most interesting result is that PDX models from gastric cancer (GC) had much higher rates of lymphoma formation than those from colorectal cancer (CRC). We then conducted a comprehensive investigation into the pathogenesis of this phenomenon.

## Results

**Figure 1:**
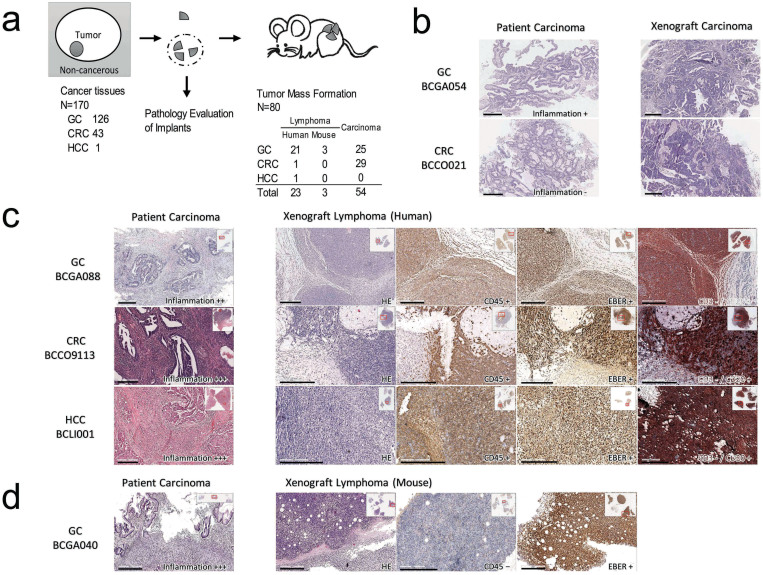
Tumor formation in the xenograft NOD/SCID mouse model in stage 1. (a) Illustration of study design. Cancer tissues from 170 patients (including 126 GC, 43 CRC and 1 HCC) were procured and then xenografted into NOD/SCID mice after a small portion was set aside for pathological evaluation. In total, 80 cancer models were established and serially re-engrafted to maintain tumors in vivo. These models were finally shown to consist of 53 patient-derived carcinomas, 23 patient-derived lymphomas and 3 mouse-derived lymphomas. (b–d) Tumor formation in the xenograft NOD/SCID mouse model. (b) the representative morphology of GC and CRC patient-derived carcinomas formed in mice, and (c) 3 representative cases for each cancer type (GC, CRC and HCC) from 23 human-derived lymphoma. All cases were identified with typical B cell type morphology, hCD45 positive, CD3-/CD20+ and were EBER positive. (d) Three morphologically similar cases of lymphoma that are hCD45 negative, finally shown to be mouse-derived lymphoma by negative amplification of human gene ALU (see Supplementary Table 1). Only 1 of the 3 cases were EBER+. Scale bars, 300 μm.

For our initial attempts to generate xenografts from a variety of human cancer tissue specimens, we procured cancer tissues from 170 patients, including 126 gastric cancer (GC), 43 colorectal cancer (CRC) and 1 hepatocellular carcinoma (HCC), from consecutive patients from 2011–2012, which were then xenografted into NOD/SCID mice. Before the implantation, a small portion of the procured specimen was put aside and fixed for pathological evaluation (stage 1, Figure 1a).

Over a mean time of 4–6 months, a total of 80 human primary tumor xenograft models were established and serially re-engrafted to maintain tumors in vivo. The clinicopathological features of these patients and matched models are shown in Supplementary Table 1. Routine pathology inspections of the established tumor were conducted. Most xenografts kept the morphology of their parent cancer tissues (Fig. 1b). Unexpectedly, we observed that a very high percentage (26 of 80, 32.5%) of established xenograft models did not resemble carcinoma but instead exhibited the morphological characteristics of lymphoid neoplasms. Immunohistochemistry staining with human CD45 (hCD45) showed that 23 of the 26 lymphomas were positive, suggesting that these lymphomas were derived from the parent tumor tissues of the patients. According to hematoxylin-eosin staining, the morphology of these lymphomas showed characteristics of the B cell type and were composed of a high density of large polymorphic neoplastic cells and scattered small lymphocytes and plasma cells. The pattern of infiltration of the tissues affected is vaguely nodular or diffuse. Geographic necrosis is a prominent feature in all cases. Further CD3/CD20 double staining proved that these tumors are all CD3- and CD20+, consistent with B cell type lymphoma (Fig. 1c). The remaining 3 xenografts were hCD45 negative and were morphologically smaller cells (Fig. 1d) compared with human-derived inflammatory cells. These xenografts were further proven to be mouse-derived by negative amplification of a human gene ALU (Supplementary Table 1) and to be of mouse B-cell origin with CD3- and CD20+ (supplementary Fig. 2). We also analyzed other markers, including the gastric and colorectal tumor markers CD44 and CD133. Immunohistochemistry staining with human CD44 and CD133 antibody showed that all cases were negative (supplementary figure 3), which suggested that the identified lymphomas were not derived from gastric and colorectal tumor cells.

**Figure 2:**
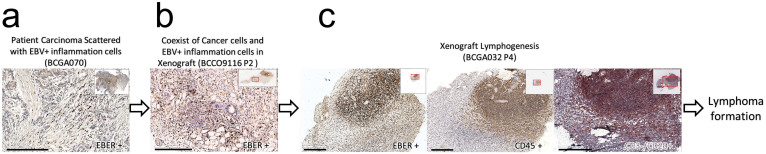
A hypothetical process of lymphomagenesis based on observation in different cases. (a) The original cancer tissues may harbor a small quantity of EBV-infected inflammation cells (BCGA070). (b) These EBV-infected inflammation cells multiply in immunodeficient NOD/SCID mice, even with the tumor cells (BCCO9116P2) and (c) may ultimately form a solid tumor consist of lymphoma cells (BCGA032P4). Scale bars, 300 μm.

We hypothesized that the observed lymphoid neoplasm was most likely associated with EBV. Because EBV is common in the pathogenesis of lymphoproliferative disorders in immunocompromised humans^15^, we evaluated our xenografts for evidence of EBV infection by in situ hybridization (ISH) for EBV-encoded RNA (EBER) and found that all the human-derived lymphomas were EBER positive. This result suggests that EBV infection is the main cause of immortalization for these inflammatory cells. However, after determining the presence of EBV in the parent tumor specimens by EBER ISH, we found that only one (parent tissue of model BCGA070, Fig. 2a) of the 23 cases was observed to have scattered EBV-positive inflammation cells. A hypothetical pathogenesis of EBV-driven lymphomagenesis in immunodeficient mice can be inferred from observation of several examples: at earlier passages, EBV-infected inflammation cells multiplied and co-existed with tumor cells, as in the model BCCO9116P2 (Fig. 2b), eventually outgrew the carcinoma cells and began to form a lymphoma tumor mass surrounded by mouse inflammatory cells, as in BCGA032P4 (Fig. 2c).

For the mouse-derived lymphoma, EBER was positive in only 1 of the 3 xenografts (Fig. 1d), which suggests that EBV might not play the most critical role in the formation of the mouse-derived lymphoma.

The interesting finding of our study is that the PDX models derived from gastric cancer had much higher rates of lymphoma formation than did those derived from colorectal cancer. In fact, although the take rate for GC was lower than that for CRC, with 25/126 (19.8%) GC tissue inoculation successes vs 29/43 (67.4%) CRC tissue inoculation successes, the lymphoma formation rate was dramatically higher in GC. To be more specific, 24 of the 126 GC cases (19.0%) formed lymphoid neoplasm; among them, 21 were human-derived, and 3 were mouse-derived. In comparison, only 1 of the 43 (2.3%) CRC cases was found to be lymphoma (Fig. 1a).

To examine the exact factors that may be related to this lymphogenesis, we performed statistical analyses of the association between xenograft phenotypes (human-derived lymphoma or carcinoma) and the clinicopathological features of the corresponding cancer. As shown in Table 1, the cancer type (OR_GC vs. CRC_ = 24.4, 95% CI: 3.1–194.2), preoperative chemotherapy (OR_yes vs. no_ = 4.6, 95% CI: 1.6–13.0) and inflammation in parent cancer (OR_++~+++ vs. −~+_ = 3.4, 95% CI: 1.2–9.6; the inflammation grade is based on the updated Sydney system for grading of gastritis^16^) were associated with an increased risk of having a human-derived lymphoma in the xenograft. Further, in the multivariate model in which all the listed potential factors were included simultaneously, only the cancer type (adjusted OR_GC vs. CRC_ = 30.0, 95% CI: 2.6–341.3) and inflammation in parent cancer (adjusted OR OR_++~+++ vs. −~+_ = 4.0, 95% CI: 1.1–14.4) were statistically significant.

**Figure 3:**
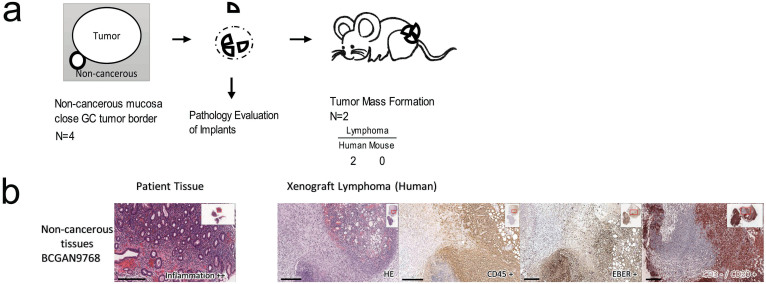
The implantation of non-cancerous mucosa with gastritis from GC patients into NOD/SCID mice in stage 2. (a) The illustration of the implantation process. Human-derived lymphomas were found to have formed in 2 cases. (b) The tumors formed in mice are also B-cell lymphomas which are hCD45+, CD3-/CD20+, and EBER+. Scale bars, 300 μm.

To validate that the cases of lymphoma formation in GC may be the consequence of the reactivation of latent EBV in remarkable intratumoral gastritis following xenotransplantation, we implanted non-cancerous tissues that were procured adjacent to the GC tumor border from 4 gastric cancer patients into NOD/SCID mice (stage 2, Fig. 3a). The results showed that two of the four mice formed human-derived lymphomas expressing hCD45+ and a B-cell subtype (CD3-/CD20+) and were positive for EBV. Pathological inspection also demonstrated that these two original parent non-cancerous tissues exhibited obvious inflammatory infiltration (Fig. 3b).

## Discussion

The implications of our results are very important to the establishment of patient-derived xenograft animal models. It is well known that EBV infects over 90% of the human population and remains latent for the lifetime of the host^17^. In the case of xenotransplantation, the small amount of latent EBV might multiply without immunosurveillance in the immunodeficient mice. In clinical settings, patients with HIV/AIDS or those receiving immunosuppressive drugs are at high risk for developing B-cell lymphomas^18^. Without effective immunosurveillance, EBV can efficiently infect B lymphocytes and transform them into a proliferative state and eventually form lymphoma^19^. Our experience with the xenotransplantation of human tumor tissues into immunodeficient mice might be a sample of the above-mentioned clinical scenarios. Therefore, the established patient-derived xenograft tumor models in immunodeficient mice should be validated by leukocyte markers such as hCD45 to exclude human-derived lymphoma. In addition, amplification and/or sequencing of specific human genes such as ALU is recommended to exclude the possibility that the tumor is of mouse origin (hCD45-). We also found that NOD/SCID mice were particularly vulnerable because we did not find any lymphoid neoplasms in our previously established 22 BALB/c xenograft nude mouse models, which were generated from 42 gastric cancer and 12 colorectal cancers (data from 2010, not shown). Our studies also showed that once lymphoma was formed in NOD/SCID mouse model, it could survive serial passages in the BALB/c nude mouse model (data not shown).

This raises the important and interesting question of why gastric cancer (GC) xenografts had much higher rates of lymphoma formation than did colorectal cancer (CRC). This finding can be explained by two facts. First, a higher extent of baseline inflammation (over 80% with H. pylori related gastritis^20^) is usually present with GC xenografts compared with primary CRC. Second, preoperative chemotherapy often aggravates inflammatory infiltration following its antitumor cytotoxicity^21^. Thus, the extent of inflammatory infiltration in inoculated parent tissue tissues such as gastric cancer is most likely directly associated with the lymphomagenesis observed in NOD/SCID mouse model.

In summary, we present here a comprehensive investigation of the EBV-related lymphoma formation in cancer patient-derived xenografts. Our results show that the extent of inflammatory infiltration in inoculated parent cancer tumor tissues is associated with EBV-driven lymphomagenesis in NOD/SCID immunodeficient mice. To better utilize patient-derived xenograft models to evaluate anticancer therapeutics, our findings suggest that the established xenograft tumor model in an immunodeficient mouse model should be validated by both leukocyte markers and human gene signatures, particularly for cancer types with remarkable baseline inflammation, such as gastric cancer. Xenografting purified or sorted tumor cells to excluded potential EBV-infected leukocytes from cancer tissues with baseline inflammation should be considered the method of choice to avoid this problem.

## Methods

### Patient tumor samples and engraftment in immunocompromised mice

Freshly and surgically removed tumor tissues were obtained from the primary cancer patients in 2011 and 2012 in Peking University Cancer Hospital. This investigation was approved by the Institutional Review Boards of the hospital, and informed consent was obtained from each patient. The cancer type, pre-operative chemotherapy status, histological grade, and vascular invasion were obtained from clinical and histopathological reports. The stage of GC was classified according to the 7th edition of the tumor-node-metastasis (TNM) classification recommended by the International Union Against Cancer. A number of patients were treated by preoperative chemotherapy or radiotherapy following the clinical practice guidelines.

Patient tumor fragments were subcutaneously engrafted into immunocompromised mice. Briefly, after removing and fixing a small portion for further pathological evaluation, the remaining tumor was sliced into 3 × 3 × 3 mm^3^ fragments and inoculated subcutaneously on the flank of mice (NOD/SCID, 6- to 8-week-old female mice, Beijing HFK Bioscience Co., Beijing, China). Tumor growth was monitored weekly using a caliper, and tumor volumes were calculated using the formula: V = 0.5 × (a × b^2^), in which a and b are the long and short diameters of the tumor, respectively. After established in mice, fragments were frozen in DMEM including 10% dimethyl sulfoxide and 40% fetal bovine serum. The established tumor models, called passage 1 or P1, were serially re-engrafted to maintain tumors in vivo. These subsequent passages were called P2, 3, 4… (<10). When the tumor sizes reached 500–1000 mm^3^, they were harvested for the next round of engraftment for serial passage or conducting studies of pharmacology, histopathology, immunohistology, cellular and molecular analysis. All procedures were under sterile conditions at BeiGene's SPF facility and conducted in accordance with the Institutional Animal Care and Use Committee (IACUC) of BeiGene.

### Pathology assessment inflammation

Tissues were fixed in 10% neutral buffered formalin and embedded in paraffin (FFPE) per standard histological procedures. After sections had been obtained from each FFPE tissue sample, histopathological evaluation was conducted using hematoxylin-eosin (HE) staining and immunohistochemical (IHC) staining. Tissue sections were digitally imaged using the Aperio ScanScope XT (Aperio Technologies, Vista, CA). Two pathologists (Y.L. and Z.L.), who were blinded to any clinical information, jointly examined all the specimens, reaching a consensus on the score of each histological variable.

To grade inflammation in the tissues, multiple histology sections (4-μm-thick) were obtained from each paraffin block. Sections were stained with HE. Because the majority of our cancer cases were gastric cancer, the inflammation grade was based on the updated Sydney system for gastritis^16^. Visual analogue scales (− = absent; + = mild; ++ = moderate; +++ = severe) were used as a reference in grading mucosal and inflammatory infiltration in each slide. (See Supplementary Fig. 1)

### Immunohistochemical staining

Standard immunohistochemistry (IHC) was used to analyze tissues from the PDX xenograft models and parent tumors. Briefly, after deparaffinization and rehydratation of 4-μm-thick tissue sections, endogenous peroxidase activity was blocked with 3% hydrogen peroxide for 15 minutes at room temperature. After pressure cooking the slides in 10 mmol/L EDTA (pH 8.0) for 3 minutes, the sections were blocked with 10% normal serum from the secondary antibody species and then incubated for 1 hour as follows: monoclonal mouse anti-human hCD45 antibody (IR75161, DAKO), CD44 antibody (ab9524, Abcam), CD133 (130-090-422, miltenyi biotec), mouse anti-human CD20/CD3 double staining cocktail working solution (Kit-8803, Maxim Biotech, Fuzhou, China). Positive staining was detected using a Detection System HRP Polymer Kit (Lab Vision, Fremont, CA, USA). DAB was used as the chromogenic substrate, and sections were counterstained with Gill's hematoxylin (Fisher Scientific, Fair Lawn, NJ). The test specimens were then scored independently by two pathologists (Y.L. and Z.L.) in a blinded fashion.

### In situ hybridization for EBER

To detect EBV infection, chromogenic in situ hybridization (CISH) assays were performed on 4-μm-thick sections using a Bond Max™ autostainer (Leica Microsystems, Germany), according to the manufacturer's instructions. Cells containing EBER transcripts were evaluated as positive when an intense, brown, predominantly nuclear staining was present in cells.

### Amplification of human ALU gene

One piece (approximately 1 × 1 × 1 mm^3^) of tumor fragment was obtained, to which 200 μl lysis buffer from Invitrogen PureLink Genomic DNA Kits (Invitrogen, Cat#: K1820-02) was added. Homogenization of tumor tissues was performed in a MP homogenization unit (Fast prep-24) (MP bio, Cat# 6004.2) at a speed setting of 6 for 60 s. The sample was centrifuged at full speed, and the supernatant was transferred to a new sterile tube. The DNA was then extracted using Invitrogen PureLink Genomic DNA Kits (Invitrogen, Cat#: K1820-02). The DNA concentration was quantified using Nanodrop and adjusted to 5 ng/μl for Q-PCR detection. The samples were analyzed for human ALU to determine whether the tumor taken from mice was of human origin using the SYBR Green Q-PCR method in a 384-well plate. The ALU primer was synthesized at Invitrogen, up: 5′ CGAGGCGGGTGGATCAT -GAGGT 3′; dn: 5′ TCTGTCGCCCAGGCCGGACT 3′. Real-time PCR was performed in the ABI 7900 real-time PCR system (Applied Biosystems™), using the following thermocycler program: 5 minutes of pre-incubation at 95°C followed by 40 cycles for 15 seconds at 95°C and one minute at 60°C. All reactions were conducted using SYBR Green I PCR Master Mix (ABI, Cat#: 4309155). At the end of each reaction, the cycle threshold (Ct) was manually set up at the level that reflected the best kinetic PCR parameters, and melting curves were acquired and analyzed. A Ct cutoff value of 20 was used to identify human DNA. The successful amplicons were verified to be consistent with human ALU sequences by sequencing.

### Statistical analysis

Odds ratios and 95% confidence intervals were estimated using univariate and multivariate logistic regression to evaluate the association between xenograft type (lymphoma or carcinoma) and a series of potential factors including age, gender, cancer type, pre-operative chemotherapy, TNM stage, vascular invasion and inflammation in parent tumor. In the multivariate model, all the listed variables were included at once. Statistical analysis was conducted using the Stata 11.2 software for Windows (College Station, TX: StataCorp LP). All P-values were two-sided, and P < 0.05 was considered statistically significant.

## Author Contributions

L.Z. and J.J. conceived and designed the experiments; L.Z., X.W., Z.T., S.L., Y.H., X.Z., X.W., Z.B., A.W., Z.L., Y.L., D.S. and L.W. performed the animal model construction and part of the pathologic experiments; Y.L., Z.L., X.H., L.J. and Q.K. performed the pathological experiments. L.Z., Y.L., X.W., Z.T. and S.L. analyzed the data and contributed to writing and editing the manuscript; L.Z., L.L. and J.J. supervised the project and wrote the manuscript.

## Supplementary Material

Supplementary Information Supplementary information

## Figures and Tables

**Table 1:** Crude and adjusted odds ratio of the clinicopathological factors of the parent cancer related to the lymphoma formation

		Xenograft Carcinoma	Xenograft Human Lymphoma	Crude OR (95% CI)	Adjusted OR (95% CI)
Age					
	<60	26	13	1.0	1.0
	≥60	28	9	0.6(0.2–1.8)	0.5(0.1–1.7)
Sex					
	Female	20	4	1.0	1.0
	Male	34	18	2.6(0.8–8.9)	2.8(0.5–15.3)
Cancer type					
	CRC	29	1	1.0	1.0
	GC	25	21	24.4(3.1–194.2)	30.0(2.6–341.3)
Pre-operative chemotherapy					
	No	39	8	1.0	1.0
	Yes	15	14	4.6(1.6–13.0)	1.0(0.2–4.6)
TNM stage					
	I/II	19	9	1.0	1.0
	III/IV	35	13	0.8(0.3–2.2)	1.0(0.2–6)
Vascular Invasion in parent tumor					
	−	36	13	1.0	1.0
	+	18	9	1.4(0.5–3.8)	0.8(0.2–4.6)
Inflammation in parent tumor					
	−/+	33	7	1.0	1.0
	++/+++	21	15	3.4(1.2–9.6)	4.0(1.1–14.4)
