# Supplementary material for: The extent of inflammatory infiltration in primary cancer tissues is associated with lymphomagenesis in immunodeficient mice
Source: Sci Rep. 2015 Mar 30;5:9447. doi: 10.1038/srep09447 (PMC4377553; doi:10.1038/srep09447)

**Title**

The extent of inflammatory infiltration in primary cancer tissues is associated with lymphomagenesis in immunodeficient mice

**Authors**

Lianhai Zhang, Yiqiang Liu, Xiaohong Wang, Zhiyu Tang, Shuangxi Li, Ying Hu, Xianglong Zong, Xiaojiang Wu, Zhaode Bu, Aiwen Wu, Ziyu Li, Zhongwu Li, Xiaozheng Huang, Ling Jia, Qiang Kang, Yong Liu, David Sutton, Lai Wang, Lusong Luo and Jiafu Ji

**Supplementary Table 1.** The clinicopathological feature of patients and matched xenograft models in stage 1 (n=80) and stage 2 (n=2).

**Supplementary figure 1**

Inflammation grade in cancer tissues. The inflammation status in the tissues is evaluated based on the updated Sydney system. Visual analogue scales (- = absent; + = mild; ++ = moderate; +++ = severe) were used as reference in grading inflammatory infiltration. Scale bars, 300μm.


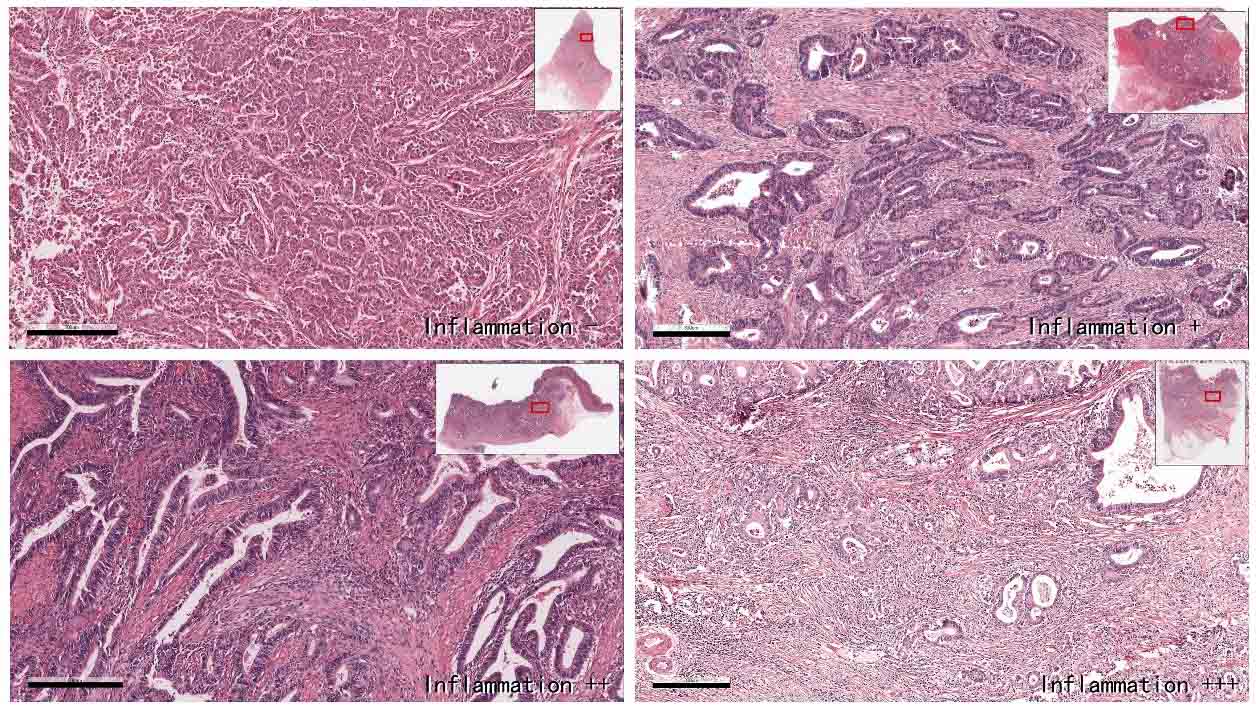


**Supplementary figure 2**

Three of twenty-six mouse-derived lymphoid neoplasms were proved to be of mouse B-cell derived with CD3-, CD56- and CD20+. Amplification x200.


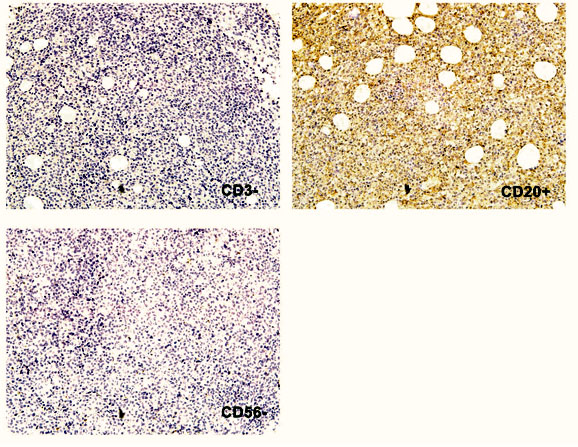


**Supplementary figure 3**

All of the mouse-derived lymphoid neoplasms were CD44 negative and CD133 negative. Amplification x200.


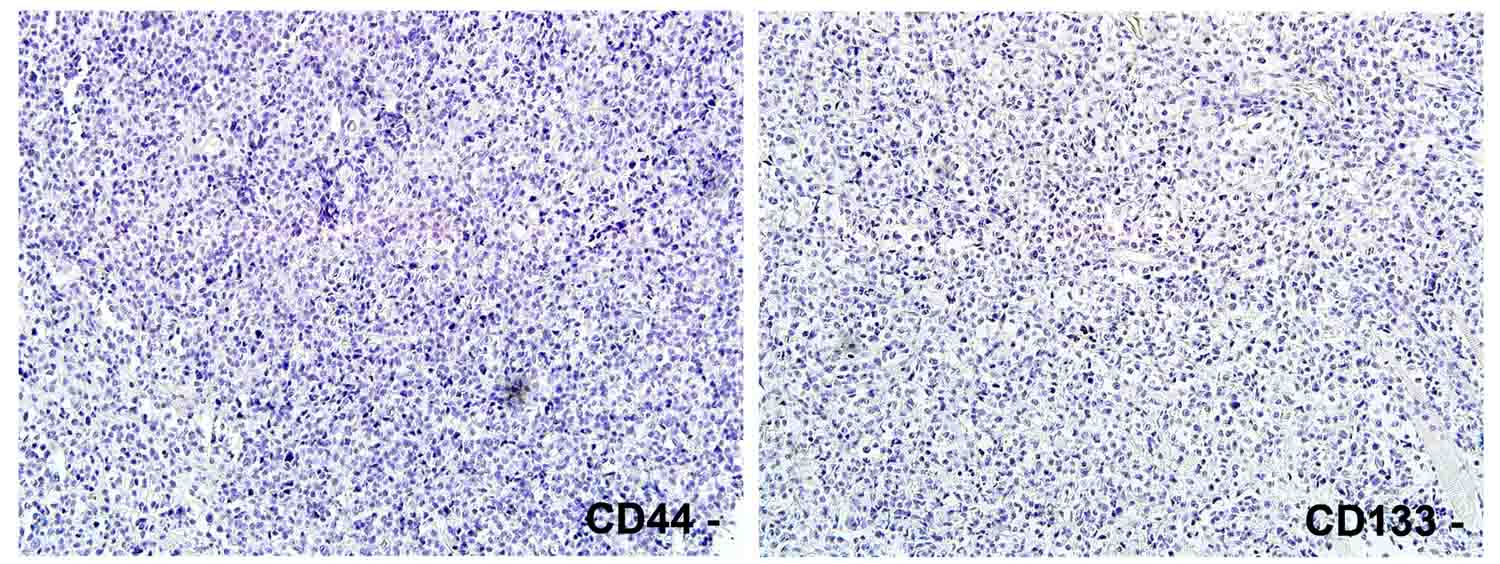

Supplement: Supplementary Information [file srep09447-s1.doc]
